# Next-generation clinical sequencing in a children’s hospital

**DOI:** 10.1186/gb-2011-12-s1-i9

**Published:** 2011-09-19

**Authors:** Stephen Kingsmore

**Affiliations:** 1 Children’s Mercy Hospital, Kansas City, MO, USA

## 

Next-generation sequencing and analysis tools are reaching the mature stage at which mentioning their usefulness for clinical testing is not an oxymoron. Indeed, next-generation clinical sequencing has the potential to transform children’s health care, because inherited illnesses account for much of the childhood disease burden. I will discuss the first year of the integration of genomic medicine for Mendelian diseases at Children’s Mercy Hospital.

